# A Technology-Based Physical Activity Intervention for Patients With Metastatic Breast Cancer (Fit2ThriveMB): Protocol for a Randomized Controlled Trial

**DOI:** 10.2196/24254

**Published:** 2021-04-23

**Authors:** Siobhan Phillips, Payton Solk, Whitney Welch, Lisa Auster-Gussman, Marilyn Lu, Erin Cullather, Emily Torre, Madelyn Whitaker, Emily Izenman, Jennifer La, Jungwha Lee, Bonnie Spring, William Gradishar

**Affiliations:** 1 Northwestern University Feinberg School of Medicine Chicago, IL United States

**Keywords:** physical activity, metastatic breast cancer, technology, randomized controlled trial, mobile phone

## Abstract

**Background:**

Increased incidence and life expectancy have resulted in a growing population of patients with metastatic breast cancer, and these patients experience high rates of morbidity and premature mortality. Increased physical activity (PA) is consistently associated with improved health and disease outcomes among early-stage survivors. However, there is a paucity of research on PA in patients with metastatic breast cancer, and existing PA interventions have exhibited low feasibility because of their focus on intense PA and/or requirement of on-site visits. Mobile health (mHealth)–based PA interventions may be particularly useful for patients with metastatic breast cancer because they allow for remote monitoring, which facilitates individual tailoring of PA recommendations to patients’ abilities and may minimize participant burden. However, no studies have examined mHealth PA interventions in patients with metastatic breast cancer.

**Objective:**

We aim to address these critical research gaps by testing a highly tailored technology-based intervention to promote PA of any intensity (ie, light, moderate, or vigorous) by increasing daily steps in patients with metastatic breast cancer. The primary aim of this study is to test the feasibility and acceptability of the Fit2ThriveMB intervention. We will also examine outcome patterns suggesting the efficacy of Fit2ThriveMB on symptom burden, quality of life, and functional performance.

**Methods:**

The Fit2ThriveMB trial is a two-arm pilot randomized controlled trial that will compare the effects of a smartphone-delivered, home-based PA intervention and an attention-control education condition on PA and quality of life in low-active female patients with metastatic breast cancer. A subsample (n=25) will also complete functional performance measures. This innovative trial will recruit 50 participants who will be randomized into the study’s intervention or control arm. The intervention will last 12 weeks. The Fit2ThriveMB intervention consists of a Fitbit, coaching calls, and the Fit2ThriveMB smartphone app that provides self-monitoring, a tailored goal-setting tool, real-time tailored feedback, app notifications, and a group message board. Assessments will occur at baseline and post intervention.

**Results:**

The Fit2ThriveMB study is ongoing. Data collection ended in February 2021.

**Conclusions:**

Data from this study will provide the preliminary effect sizes needed to assemble an intervention that is to be evaluated in a fully powered trial. In addition, these data will provide essential evidence to support the feasibility and acceptability of using a technology-based PA promotion intervention, a scalable strategy that could be easily integrated into care, among patients with metastatic breast cancer.

**Trial Registration:**

ClinicalTrials.gov NCT04129346; https://clinicaltrials.gov/ct2/show/NCT04129346

**International Registered Report Identifier (IRRID):**

DERR1-10.2196/24254

## Introduction

### Background

Almost 155,000 women live with metastatic breast cancer in the United States as of January 1, 2017 [1]. The number of women diagnosed with metastatic breast cancer is expected to increase by 31% over the next 10 years [1]. Furthermore, treatment advances have doubled the 5-year survival rates in the last two decades by 36% and 11% of women survive for ≥10 years [1]. Thus, the number of women with metastatic breast cancer is increasing. Women with metastatic breast cancer have higher rates of physical impairment [2], greater symptom burden [3], and lower levels of fitness and strength [3,4] than early-stage survivors and noncancer controls, resulting in compromised quality of life (QoL). Interventions are needed to alleviate adverse health effects and allow patients with metastatic breast cancer to function optimally in the years they survive with advanced cancer. However, few health-enhancing interventions exist for women with metastatic breast cancer, and only 2%-5% [5] of research funds for breast cancer are spent on metastatic disease despite its high morbidity rates. Thus, research in this population is urgently needed.

Increased physical activity (PA) is consistently associated with fewer treatment-related side effects, higher QoL, increased survival, and reduced recurrence and mortality among survivors of early-stage breast cancer [6-9]. Increasing light-intensity activity and reducing sedentary time may also reduce functional decline [10] and mortality [11] and improve QoL [11,12] and body composition [13] independent of more intense activity. However, there is a paucity of research on PA in patients with metastatic breast cancer. Two recent reviews of PA interventions in mixed samples with advanced cancer indicate that PA is safe and feasible for these populations, and PA may be associated with a variety of benefits, including improved aerobic capacity, strength, physical and psychosocial function, QoL, fatigue, sleep quality, and body composition [14,15]. In addition, a small study on patients with metastatic breast cancer found that increased PA was associated with longer survival [16]. However, only 4 randomized controlled trials (RCTs) have focused specifically on PA in patients with metastatic breast cancer [3,17-19]. Although these studies demonstrated that both supervised and home-based PA programs are safe for patients with metastatic breast cancer, findings regarding effects on PA, fitness, and patient-reported outcomes were equivocal; 2 studies found no effect on the outcomes of interest [17,20], and 2 found trends toward moderate intervention effects on fatigue and physical well-being, fitness, and the 6-minute walk test [18,19]. However, sample sizes were small. The null findings were largely attributed to the lack of feasibility (ie, low adherence, poor attendance at in-person sessions, high dose modification, and attrition) because of their focus on intense activity and/or requirement of on-site visits. The one study that used a wearable tracker is a single-arm study that found high adherence (96%) to using the tracker [21]. Using mobile health (mHealth) technology for PA promotion interventions may be particularly useful for these women because it allows for remote monitoring of patients, which facilitates individual tailoring of PA programs to patients’ abilities. mHealth interventions also do not require travel to on-site, supervised activity sessions, thus reducing participant burden. However, no studies have examined the effects of an mHealth intervention on objectively measured PA in patients with metastatic breast cancer in a controlled trial.

The primary goal of this study is to address these critical research gaps by testing the feasibility and acceptability of a highly tailored 12-week mHealth intervention to promote activity of any intensity (ie, light, moderate, or vigorous) by increasing daily steps using a two-arm RCT in patients with metastatic breast cancer. Secondary goals include examining the intervention’s effects compared with a control group on symptom burden and QoL in the full sample and functional performance in a subsample (n=25).

### Study Aims

The Fit2ThriveMB trial was designed to pursue 3 objectives. First, we will examine the feasibility and acceptability of Fit2ThriveMB, a 12-week mHealth PA intervention in patients with metastatic breast cancer. Second, we will examine the potential effects of Fit2ThriveMB on accelerometer-assessed PA. Finally, we will examine outcome patterns suggesting the efficacy of Fit2ThriveMB in improving symptom burden (ie, fatigue, depression, anxiety, pain, and physical function) and QoL in the full sample and functional performance in a subsample.

## Methods

### Overview and Study Design

**Figure 1:**
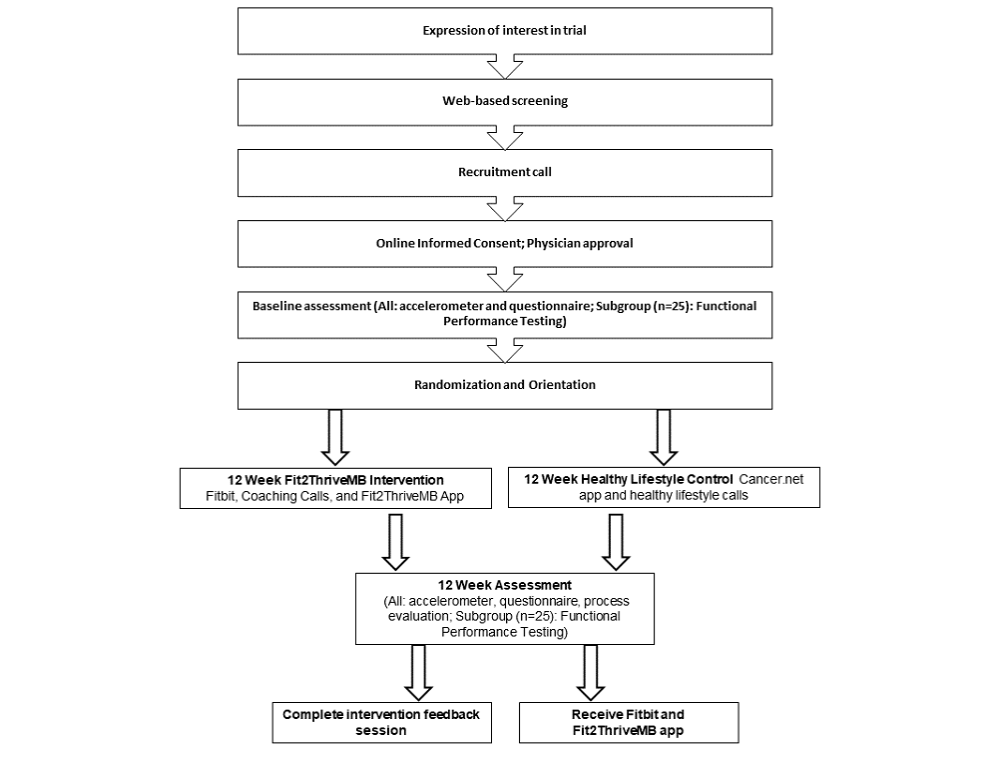
Fit2ThriveMB participants flow through the study.

FFit2ThriveMB is a 12-week mHealth pilot RCT conducted to examine the feasibility and acceptability of a 12-week technology-based PA promotion program. We will also compare the Fit2ThriveMB intervention with a healthy lifestyle attention-control group and examine outcome patterns, suggesting the efficacy of Fit2ThriveMB for increasing PA and improving symptom burden, QoL, and functional performance. We randomized 50 low-active patients with metastatic breast cancer to each condition. Participants assigned to the Fit2ThriveMB condition will receive a PA promotion intervention that incorporates behavior change principles based on social cognitive theory (SCT) [22] and includes a Fitbit, weekly coaching calls, and the Fit2ThriveMB smartphone app, which includes tools for self-monitoring and tailored goal setting, real-time tailored feedback, app notifications, and a social feed. Participants in the healthy lifestyle control will be instructed to download a commercially available cancer care app (Cancer.Net) and will be provided with educational materials and support calls. The participants will complete assessments at baseline and postintervention (12 weeks). Participants in the control condition will receive the Fit2ThriveMB app and a Fitbit following completion of the 12-week assessments. An overview of participant flow through the study is shown in Figure 1.

### Conceptual Model

**Figure 2:**
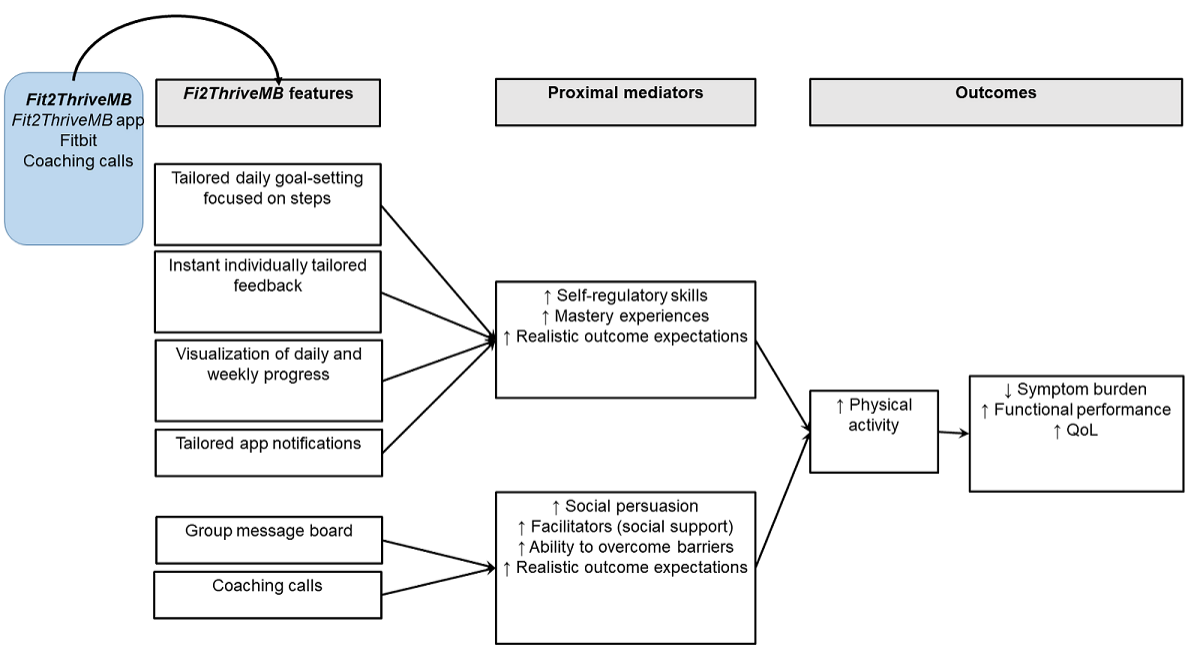
Fit2ThriveMB conceptual model. QoL: quality of life.

SCT [22] is well recognized as a useful framework for designing PA interventions for cancer survivors [23,24]. SCT specifies a core set of determinants (self-efficacy, goal setting, facilitators and barriers, and outcome expectations) and the mechanisms by which they work [25]. Specifically, SCT postulates that self-efficacy is both directly and indirectly related to PA, via facilitators and barriers (ie, lack of access to facilities or social support), goal setting and self-regulation (ie, monitoring PA or using feedback to measure progress) and outcome expectations (ie, belief that PA will result in a specific outcome) [25]. Figure 2 details our conceptual model of how we hypothesize that Fit2ThriveMB will influence PA, symptom burden, QoL, and functional performance. On the basis of our conceptual model, we hypothesize that the Fit2ThriveMB intervention components will improve self-regulatory and goal-setting skills, increase self-efficacy via increased mastery experiences and social persuasion, increase realistic outcome expectations, improve facilitators by increasing social support, and increase the ability to overcome barriers, resulting in increased PA. Increased PA is, in turn, hypothesized to result in favorable changes in symptom burden, QoL, and functional performance.

### Recruitment and Screening

#### Eligibility

The inclusion criteria are being female, aged ≥18 years, diagnosed with metastatic breast cancer (stage IV), with a life expectancy of ≥12 months, low active (ie, self-reported engagement in <150 minutes of moderate-to-vigorous physical activity [MVPA] per week), fluent in spoken and written English, owning a smartphone, and having access to a computer with internet access to complete assessments. The exclusion criteria include untreated brain metastases, uncontrolled cardiovascular disease or other major contraindications (ie, nonambulatory or severe cognitive or functional limitations) to activity participation, and current enrollment in another dietary or activity trial. All participants must obtain medical and diagnostic eligibility clearance from their oncologists. Diagnosis will also be confirmed via medical records before enrollment.

#### Recruitment Procedures

Women will primarily be recruited from oncology clinics at a large Midwestern academic medical center via medical records and physician referrals. Patients will also be recruited via support groups, fliers, and local cancer events. All potential participants will be sent a study recruitment email, providing a brief description of the study and assessing patient interest. If interested, a personalized link in the email will automatically redirect the potential participant to complete the web-based screening. Potential participants will be sent 2 recruitment emails. If no response is received to these contact attempts, after 1-2 weeks, the study team will follow up with a phone call. If the individual answers and is interested in participating, the study team will describe the study and screen the individual via phone or resend them the link to the web-based screener, depending on patient preference. If the potential participant does not answer the phone call, the study team will leave a voicemail and send 1 final recruitment email with a link to schedule a recruitment call.

#### Screening

All interested women will complete the initial eligibility screening on the web or over the phone. The screening will take approximately 15 minutes and includes an evaluation of all eligibility criteria, including self-reported PA and completion of the Physical Activity Readiness Questionnaire [26], which assesses cardiovascular disease history, symptoms, risk factors, and other health issues. All participants must obtain medical and eligibility clearance from their oncologist to participate. Diagnosis will also be confirmed via medical records before enrollment. The subsample of participants who participate in functional performance tests will also be asked to complete the Falls Risk Questionnaire [27], and the results will be shared with the oncologist to inform clearance decisions and used by the study staff to prepare for any potential balance issues that may arise during the functional performance test. Eligible candidates will be emailed a copy of the informed consent and a study overview document to review and will be scheduled for a recruitment call.

#### Recruitment Call

During the recruitment phone call, staff will explain the study in greater detail and confirm that participants meet the eligibility criteria for cancer diagnosis, their activity level and if they have a major contraindication to PA participation, and their intention to participate in the study. The call will last, on average, from 15 minutes to 20 minutes. If the potential participant is screened over the phone, the recruitment call will be completed as part of the screening phone call unless the individual prefers to complete it at a later date. Interested and eligible women will be emailed a link to a web-based version of the informed consent and a copy of the permission to contact the physician form. They will be instructed to complete and sign both documents and submit them on the web. Once both documents are complete, the study team will acquire physician consent.

### Data Collection

All participants who consent to participate will be shipped an assessment kit. The kit will include an accelerometer, accelerometer instructions, and an accelerometer log for all participants. Participants will be instructed to wear the accelerometer for 7 consecutive days (see *Physical Activity Measures* for further details) and return the accelerometer to the study team via the provided postage-paid return envelope. A personalized link to a battery of questionnaires will be emailed to participants. The participants will complete the questionnaires at home and submit them on the web via RedCap before their videoconference. These procedures will be the same at both testing time points.

For the subgroup participating in the functional performance test, the assessment kit will also include a functional performance test kit with instructions for preparing for their videoconference functional performance testing at baseline (see *Physical Function Measures* for further details). Following baseline functional performance testing, participants will be instructed to put the functional performance test kit in a safe-keeping spot to reuse at 12 weeks. After the 12-week testing, they will be instructed to send the test kit materials back.

Participants will be regularly reminded via phone or email to wear the accelerometer, answer the web-based questionnaires, and attend their functional performance testing sessions. Participants will not be randomized until all the baseline data are complete. Participants will be incentivized for completion of assessments and allowed to keep the Fitbit (intervention) or provided with a Fitbit (control) if they complete assessments at both time points.

All Fitbit data and data on app usage will be collected throughout the duration of the intervention and will be stored in a database developed specifically for this study, which is on a secure, password-protected server only accessible to study investigators.

### Measures

Adherence and retention will be evaluated during the intervention. All other measures will be assessed at baseline and postintervention (12 weeks).

#### Feasibility and Acceptability

The feasibility of each component will be measured during the intervention and immediately after the intervention. Measures include participant retention (number of participants who dropped out/number randomized), objective (when possible) and self-reported usage of intervention components (percentage of days of adhering to the daily goal and percentage of days of wearing Fitbit), and safety as measured by the number and severity of adverse events reported spontaneously and during nonspontaneous assessments. Fitbit is sufficiently validated for steps to support its role as an adherence measure in this study. Adherence during the 12-week intervention will be monitored continuously using the Fit2ThriveMB app. We will obtain Fitbit wear time, steps, and time spent in sedentary, and light-, moderate-, and vigorous-intensity activity. Acceptability will be measured via a process evaluation of the perceptions of experiences of patients with metastatic breast cancer with Fit2ThriveMB*.* This evaluation will assess the following: perceived effectiveness of intervention components, plans to continue PA and intervention use, intervention elements liked or disliked, and satisfaction with program delivery, assessments, and staff. Items will be rated on a scale ranging from 1 (worst) to 5 (best). We will also conduct semistructured postprogram interviews with individuals who participate in the intervention group to obtain feedback on each intervention component and recommendations for how the intervention can be improved*.*

#### Physical Activity

PA will be measured objectively using self-report measures. PA will be assessed objectively by accelerometry at baseline and 12 weeks using an ActiGraph accelerometer (model GT3X-BT, ActiGraph). ActiGraph, a valid and reliable measure of PA [28,29], has been widely used in oncology research [30,31]. Participants will be instructed to wear the activity monitor on the nondominant hip during all waking hours (except when bathing or swimming) for 7 consecutive days. The accelerometer will collect activity data in 10-second intervals (ie, epochs). Upon receipt of the accelerometer, the data will be immediately downloaded and checked for valid wear time. If there is not at least five days of valid measurement with 10 hours of valid wear time [32], participants will be asked to rewear the monitor. The average number of minutes of daily total activity and time spent in sedentary, light, moderate, and vigorous activity will be calculated using established cut-points [33,34]. Data on steps will also be collected. We are primarily interested in data on the total minutes of PA of any intensity (total weekly light, moderate, and vigorous) and steps but will also examine average daily total counts. The Godin Leisure-Time Exercise Questionnaire [35] will be used to assess self-reported minutes of PA (light, moderate, and vigorous) during each measurement period.

Fitbit will be used to collect activity data throughout the intervention, and participants will wear both Fitbit and ActiGraph during week 12 assessments. Fitbit data will not be used as an outcome measure. Instead, these data will be used to monitor adherence, as detailed below.

#### Symptom Burden

Women will complete reliable, well-validated Patient-Reported Outcomes Measurement Information System [36] self-report assessments of symptoms including fatigue, depression, anxiety, pain interference, and physical function, and the Functional Assessment of Cancer Therapy-Breast [37], a well-validated measure of QoL, via web-based questionnaires at each time point. The details of these measures are provided in Table 1.

**Table 1:** Study questionnaires.

Construct and measure			Description
**Symptom burden [36]**			
	PROMIS^a^ Physical Function Short Form 20a [36]		Measures functional limitations and interference over the past 7 days
	PROMIS Fatigue Short Form 8b [36]		Measures frequency of fatigue symptoms and interference over the past 7 days
	PROMIS Depression Short Form 8b [36]		Measures the frequency of a variety of depressive symptoms over the past 7 days
	PROMIS Anxiety Short Form 8b [36]		Assesses self-reported fear (fearfulness and panic), anxious misery (worry and dread), hyperarousal (tension, nervousness, and restlessness), and somatic symptoms related to arousal (racing heart and dizziness) over the past 7 days
	PROMIS Pain Interference Short Form 8b [36]		Measures the self-reported consequences of pain on relevant aspects of one’s life over the past 7 days
**Quality of life**			
	Functional Assessment of Cancer Therapy-Breast [37]		Assesses participants’ physical, social, family, emotional and functional well-being, and breast cancer–specific concerns
**SCT^b^ constructs**			
	**Self-efficacy**		
		Exercise Self-Efficacy Scale [38]	Assesses beliefs in ability to be physically active over the next 12 weeks
		Barriers Self-Efficacy Scale [38]	Assesses beliefs in ability to be physically active over the next 12 weeks despite common barriers
	**Outcome expectations**		
		Multidimensional Outcome Expectations for Exercise Scale [39]	Assesses social, self-evaluative, and physical outcome expectations for physical activity
	**Goal** **setting**		
		Exercise Goal-Setting Scale [40]	Assesses physical activity–related goal setting, self-monitoring, and problem solving
	**Facilitators and barriers**		
		Social Support for Exercise Scale [41]	Measure physical activity support received from friends, family, and other survivors
		Physical Activity Enjoyment Scale [42]	Measures enjoyment and satisfaction with current physical activity program

^a^PROMIS: Patient-Reported Outcomes Measurement Information System.

^b^SCT: social cognitive theory.

#### Functional Performance

Originally, the entire sample was used to complete functional performance measures in-person at each time point. However, as a result of the 2020 coronavirus global pandemic, these measures will only be completed on a subset (n=25) of the sample at both time points via a videoconference. Enrollment began in January 2020, and 25 women were enrolled in March 2020. These initial 25 participants completed in-person functional performance testing at baseline but were unable to complete 12-week functional performance testing because in-person research was suspended due to the coronavirus pandemic. Thus, no 12-week functional performance data were collected for the first 25 participants. However, functional performance data will be collected remotely from the next 25 participants to enroll.

Participants will complete the Short Physical Performance Battery (SPPB), a well-validated physical function performance measure [43,44]. The SPPB score is based on timed measures of gait speed, the ability to rise from a chair, and standing balance. Gait speed will be measured using the faster of 2 recorded times over a 4-m course. Chair stand time will be measured as the time needed to rise 5 times from a seated position in a chair, with arms folded across the chest. For the balance test, participants will be asked to maintain their feet side-by-side in semitandem and tandem positions for 10 seconds each. Each individual performance measure will be scored according to established cut-points [43] and aggregated for a total SPPB score. We will also use several items from the Senior Fitness Test [45], including the 8-foot up-and-go test, a test of physical agility and dynamic balance; the Arm Curl test, which assesses arm muscle strength endurance, specifically of the biceps; and the 2-minute step test, an aerobic endurance test, which counts the number of full in-place steps completed in 2 minutes. Participants will also complete a 30-second one-leg stand test on each side (right and left), where they will be instructed to stand on 1 foot for up to 30 seconds and timed. Finally, participants will complete a 6-minute walk test [46] to assess their submaximal level of functional capacity.

Participants will be mailed a functional performance test tool kit that will include all of the supplies necessary to complete the functional performance tests. The study staff will administer functional performance testing via videoconference using a presentation with prerecorded videos detailing test set up and instructions. Each testing session will take approximately 60-90 minutes; 2-3 staff members certified in testing procedures will be present at the videoconference, varying depending on the testing time point. A staff member will administer the tests and be blinded to the condition; the other staff members will support the administrator and assist with any technology issues or in the event of an emergency. This staff member may or may not be blinded but will have no previous relationship (ie, not assigned to support coaching calls) with the participant. To ensure safety, participants will be asked to have another individual present at home during the test, if possible, and the study staff will be cardiopulmonary resuscitation or first-aid certified. In addition, the participant’s address and major cross streets will be confirmed and the nonemergency police number will be written in the participant file so that both study staff have access to this number in the event of an emergency during testing.

If the participant consents, the tests will be video recorded for quality control. Metrics on the feasibility of conducting these assessments via videoconference, including mailing issues and technology issues, will be recorded. Participants will be asked to complete a brief web-based survey to assess their experience with functional performance testing, including ease and satisfaction with using the videoconference software and functional performance test kit supplies, setting up testing, and following the written and videoconference instructions at each time point.

#### Fidelity and Adherence

Fidelity and adherence to each Fit2ThriveMB intervention and control group components are detailed in Textbox 1. All measures are objectively obtained unless otherwise noted. Coaching calls are recorded for all participants who consent to the audio recording.

**Measures of fidelity and adherence to intervention components.**

Fit2ThriveMB intervention

- Fitbit usage: average number of days of wearing the Fitbit (≥2000 steps/day) and proportion of days of wearing the Fitbit
- Fit2ThriveMB app: average number of days participants opened the app and average time spent using the app each day
- Daily goal setting: average number of days participants responded to the survey
- Fit2ThriveMB app social feed: total number of social feed posts; the average number of days of access to the social feed page
- Fit2ThriveMB app notifications: average number of messages read
- Fit2ThriveMB coaching calls: total percentage of coaching calls attended; average time per call; fidelity to coaching call script

Control group

- Cancer.Net usage: self-reported number of days using the app
- Support calls: total percentage of calls attended; average time per call; and fidelity to support the call script

### Additional Variables

#### Demographic and Disease Characteristics

Participants will self-report on demographic characteristics and health status, including age, height, weight, race or ethnicity, marital status, education, income, and comorbid conditions. They will also self-report disease and treatment characteristics, including date of diagnosis, site of metastasis, hormone receptor status, and treatment received. The diagnosis and treatment data will be verified using medical records.

#### SCT Constructs

All SCT constructs will be assessed, including self-efficacy, goal setting, outcome expectations, and barriers and facilitators (Table 1).

### Randomization

Participants will be randomized 1:1 to the Fit2ThriveMB intervention or healthy waitlist control using computer-generated randomly permuted blocks. They will receive their condition assignment following the completion of all baseline assessments. To prevent bias, group assignment is concealed in an opaque envelope until allocation is completed and envelopes are prepared by an individual who is blinded. The nature of the intervention precludes blinding of the staff and complete blinding of participants. However, the statistician will be blinded, and all individuals conducting functional performance testing at follow-up testing will be blinded to the group assignment. Participants will start the intervention in cohorts of 6-10 individuals to ensure an adequate number of participants for the Fit2ThriveMB social feed.

#### Study Packet

All participants will be given a study packet following randomization. Individuals assigned to Fit2ThriveMB will be provided with a Fitbit, instructions on all intervention procedures, and instructions for setting up the Fitbit and downloading, setting up, and using the Fitbit and Fit2ThriveMB apps. Individuals assigned to the healthy lifestyle control groups will be given instructions on all intervention procedures and instructions for downloading, setting up, and using the Cancer.Net app.

#### Orientation

Participants will be scheduled for an in-person or videoconference orientation specific to the intervention condition they are assigned before starting the intervention. Study staff will reiterate the expectations for the condition to which they are assigned, answer any questions, and troubleshoot any technical issues they may be experiencing.

### Intervention: Fit2ThriveMB Arm

The Fit2ThriveMB intervention arm consists of 3 intervention components described in detail below: the Fitbit, the Fit2ThriveMB app, and coaching calls. Participants will be encouraged to accumulate more steps throughout the day by (1) moving more and sitting less (ie, taking more steps and reducing the time they spend sedentary), (2) adding planned structured exercise sessions to their day, or (3) a combination of (1) and (2). They will also be provided with a more detailed exercise prescription if they decide to adopt approach (2) (Table 2). Participants will have access to the app for the duration of the 12-week study and the remaining duration of the funding period (up to approximately 12 months).

**Table 2:** Fit2ThriveMB exercise prescription.

Week	Weekly exercise goal (min)	Number of sessions per week	Session duration (min)	Estimated steps per session			Rating of perceived exertion	Heart rate target (% of maximum heart rate)
				Light intensity	Moderate intensity	Vigorous intensity		
1	30	2-3	10-15	800-1200	1000-1500	—^a^	9-12	Up to 70%
2	45	2-3	15-20	1200-1600	1500-2000	—	9-12	Up to 70%
3	60	3	20	1600	2000	—	9-12	Up to 70%
4	75	3	25	2000	2500	—	9-12	Up to 70%
5	90	3	30	2400	3000	—	9-12	Up to 70%
6	105	3	35	2800	3500	—	9-12	Up to 70%
7	120	3-4	30-40	2400-3200	3000-4000	—	9-12	Up to 70%
8	135	3-4	30-45	2400-3600	3000-4500	3900-5850	9-15	Up to 85%
9	150+	3-5	30-60	2400-4800	3000-6000	3900-7800	9-15	Up to 85%
10	150+	3-5	30-60	2400-4800	3000-6000	3900-7800	9-15	Up to 85%
11	150+	3-5	30-60	2400-4800	3000-6000	3900-7800	9-15	Up to 85%
12	150+	3-5	30-60	2400-4800	3000-6000	3900-7800	9-15	Up to 85%

^a^Vigorous cells are empty in weeks 1-7 because vigorous activity is not recommended until week 8 to ensure proper progression.

#### Fitbit

Participants will be provided with a Fitbit InspireHR. They will be asked to download the Fitbit app and wear the Fitbit 24/7 throughout the 12-week study period. Fitbit measures PA intensity, steps, and heart rate and synchronizes directly with the Fitbit app, which automatically synchronizes with the Fit2ThriveMB app and provides Fitbit data to the study team in real time.

#### Fit2ThriveMB App

**Figure 3:**
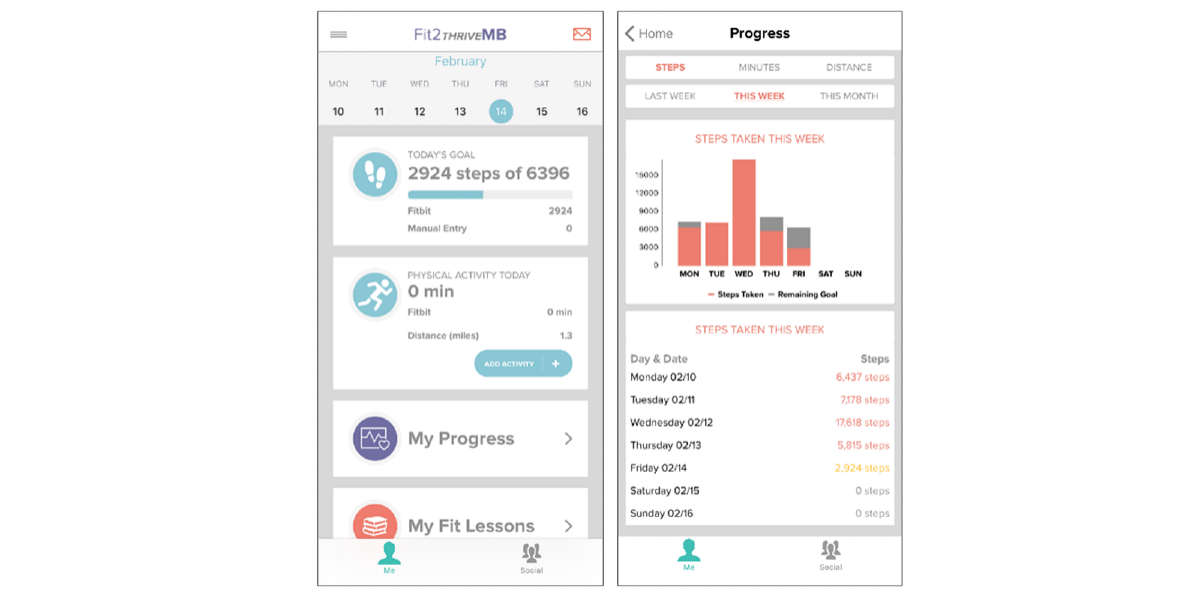
Fit2ThriveMB app home screen and fit progress.

The custom-built Fit2ThriveMB app will encourage participants to increase their PA through SCT [22] principles, including goal setting, self-monitoring, personalized feedback on progress, app notifications, education on behavior change techniques, and social support. The Fit2ThriveMB app home screen and progress screen are shown in Figure 3. The app features are detailed below.

#### Daily Symptom Reporting and Goal Setting

**Figure 4:**
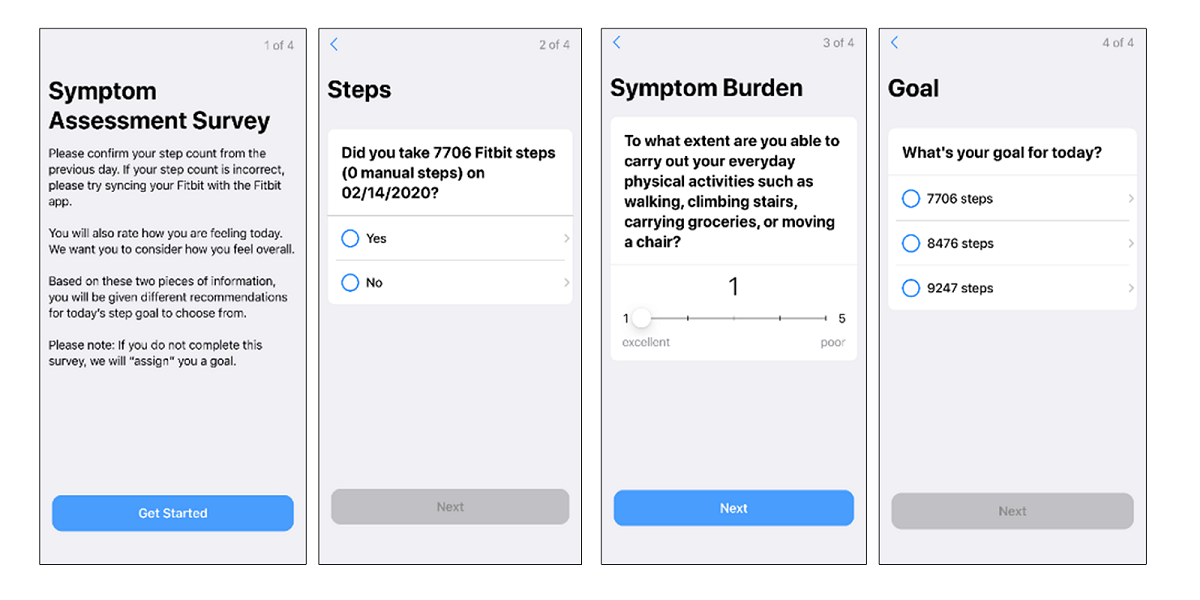
Fit2ThriveMB app symptom burden and goal-setting survey.

Participants will be prompted each morning to report the intensity of their symptom burden on a scale from 1 to 5 by answering the following question: “To what extent are you able to carry out your everyday physical activities such as walking, climbing stairs, carrying groceries, or moving a chair?” [47]. On the basis of symptom rating, whether or not they met the previous day’s step goal and/or the number of steps achieved the previous day, participants will be provided with 3-4 goal options for that day (Figure 4). The goal algorithms are presented in Table 3. Standard goal logic options of increasing or decreasing steps by 10% or 20% or remaining constant will be applied to days where the steps of the previous days are between 3751 and 12,000. As the intervention’s goal is to increase steps, the algorithm never recommends less than 3000 steps, which is considered the floor value for absolutely defined moderate-intensity walking [48]. Special goal-setting logic is applied when the Fitbit is classified as not worn (ie, <2000 steps [49]) on the previous day, steps are *low* (ie, 2001-3750) on the previous day, and steps are *high* (>12,000) on the previous day. If a participant fails to choose a goal, they are assigned the middle-level goal as their goal for the day. If a participant does not answer the symptom burden survey, their goal from the previous day is carried forward. The step goal-setting options for the first day of the intervention will be based on the average value of steps for all valid days of accelerometer data at baseline.

**Table 3:** Daily step goal logic.

Symptom rating			Step goal option 1		Step goal option 2		Step goal option 3		Step goal option 4
**Steps achieved: any value**									
	No response	—^a^		—		—		—	
**Steps achieved: no data or <2000 steps**									
	1 or 2	Steps achieved in the last available day >2000		Increase of 10%		Increase of 20%		N/A^b^	
	3	Decrease of 10%		Steps achieved in the last available day >2000		Increase of 10%		N/A	
	4 or 5	Decrease of 20%		Decrease of 10%		Steps achieved in the last available day >2000		N/A	
**Steps achieved: goal met and 2000-3750 steps**									
	1 or 2	3750		Increase of 10% (4125)		Increase of 20% (4500)		N/A	
	3	Decrease of 10% (3375)		3750		Increase of 10% (4125)		N/A	
	4 or 5	Decrease of 20% (3000)		Decrease of 10% (3375)		3750		N/A	
**Steps achieved: goal** *not met* **and 2000-3750 steps**									
	1 or 2	Decrease of 10% (3375)		3750		Increase of 10% (4125)		N/A	
	3, 4, or 5	Decrease of 20% (3000)		Decrease of 10% (3375)		3750		N/A	
**Steps achieved: goal met** **and 3751-11,999 steps**									
	1 or 2	Steps achieved in previous day		Increase of 10%		Increase of 20%		N/A	
	3	Decrease of 10%		Steps achieved in previous day		Increase of 10%		N/A	
	4 or 5	Decrease of 20%		Decrease of 10%		Steps achieved in previous day		N/A	
**Steps achieved: goal** *not met* **and 3751-11,999 steps**									
	1 or 2	Decrease of 10%		Steps achieved in previous day		Increase of 10%		N/A	
	3, 4, or 5	Decrease of 20%		Decrease of 10%		Steps achieved in previous day		N/A	
**Steps achieved: goal met and ≥12,000 steps**									
	1 or 2	Steps achieved in previous day		Increase of 10%		Increase of 20%		Decrease of 50%	
	3	Decrease of 10%		Steps achieved in previous day		Increase of 10%		Decrease of 50%	
	4 or 5	Decrease of 20%		Decrease of 10%		Steps achieved in previous day		Decrease of 50%	
**Steps achieved: goal not met and 12,000+ steps**									
	1 or 2	Decrease of 10%		Steps achieved in the previous day		Increase of 10%		Decrease of 50%	
	3, 4, or 5	Decrease of 20%		Decrease of 10%		Steps achieved in the previous day		Decrease of 50%	

^a^The goal remains the same as the last option chosen or assigned.

^b^N/A: not applicable.

#### Self-monitoring

Participants will receive feedback on their progress toward their daily step goal on the app home screen and weekly and monthly progress information in the fit progress section of the app. In addition, they can monitor progress toward their step goal on the Fitbit device screen.

#### Fit Progress

Patients will be provided with information on their steps, minutes of MVPA, and distance traveled in miles. They will be able to view this information from the previous week, current week, and current month (ie, the previous 4 weeks) of the program.

1. Steps: a bar graph displays the steps achieved for the selected period. The number of steps reached on individual days of the week is listed in the graph if the previous week or current week’s time frame is selected. If the current month is selected, the average number of steps per week is displayed.
2. Minutes: a line graph displays minutes of MVPA achieved for the selected period. Minutes of MVPA achieved on individual days of the week will be listed under the graph if the previous week or current week’s time frame is selected. If the current month is selected, the total minutes of MVPA per week are displayed.
3. Distance: a line graph displays the distance traveled in miles for the selected period. The distance traveled on individual days of the week will be listed under the graph if the previous week or current week’s time frame is selected. If the current month is selected, the total miles per week are displayed.

#### Fit Lessons

Participants will be provided with educational information on PA and effective SCT behavior change strategies to incorporate more PA into their daily lives to increase their step count (eg, 10-minute lunchtime walk, parking further from entrances, and pacing while on the phone) and structured exercise via the app home screen.

#### App Notifications

The app will send participants text messages via app notifications, including tips to increase steps, a social digest, encouragement messages, real-time PA progress messages, and reminders to rate symptom burden, explore Fit Lessons, synchronize Fitbit, and engage in the social feed. A full list of message types, frequencies, and examples is provided in Table 4. Progress messages will be tailored to each patient’s fitness PA data. Participants will be instructed to enable notifications from the Fit2ThriveMB app during download. However, due to phone carrier companies’ restrictions, we cannot require that they enable this functionality. They can also change their app notification settings to disable these messages.

**Table 4:** Fit2ThriveMB app notification types and examples.

Message type	Description	Frequency	Example
Symptom message	Reminder to complete symptom burden questionnaire	Daily within 1 hour of wake time	“Time to answer your daily survey and pick today’s step goal!”
Symptom reminder	3 reminders every 15 minutes to complete a survey	Every morning if the participant does not complete after the first message	“Reminder: complete your symptom burden survey and choose today’s step goal!”
Social cognitive theory behavior change	Message to remind participants to look at the educational materials to learn more about physical activity and healthy habits	1 time per week	“Did you know keeping a record of your activity and reviewing it can make you much more likely to reach your goals? Read more about daily tracking on the F2TMB Fit Lessons!”
Increasing steps	Educational message with ideas on how to increase daily step count by incorporating increasing steps into the daily routine	2 messages per week	“Take the stairs instead of the elevator today. Small changes like this can count toward your daily step goal!”
Rescue	Provides motivation to get in some extra steps before the day ends and reach the goal. Messages are stratified based on if they have ≥2500 steps	Daily if participant has not achieved step goal by 4 PM	“Almost there, you have [X] steps to go until you reach today’s goal. You can do it!”
Sync reminder	Reminder to sync Fitbit or add any activity for the day	Daily if the participant does not have data 1 hour before bedtime	“Remember to sync your Fitbit data and enter any other activities you did today!”
Social goal	Any participant in the group reached their step goal for that day	Up to 8 times a day (depends on groups size) if someone in the groups meets their goal	“[Z] reached 100% of her step goal today! Head to your newsfeed to congratulate her!”
Social Digest	Message consisting of a number of new posts, comments to own posts, or tags from the social digest	Every 30 minutes, if something new is posted	“There are [X] new posts and you’ve been mentioned [Y] times in your Fit2ThriveMB group!”
Reengagement	When a participant has not posted or commented in the social feed	Every 4 days, if the participant has not posted	“Your Fit2ThriveMB group members miss you! Let them know how you are doing.”
Goal progress	Provides encouraging message once 50% of the daily goal is met	Encouraging messages when transmitted Fitbit data indicate they reached 50%, 75%, and 100% of the daily step goal	“You’ve hit 50% of your step goal for the day, keep up the good work!” “Way to hit 75% of your daily goal! Only [X] steps left, let’s do this.” “100% of today’s step goal accomplished, well done!”

#### Social Feed

**Figure 5:**
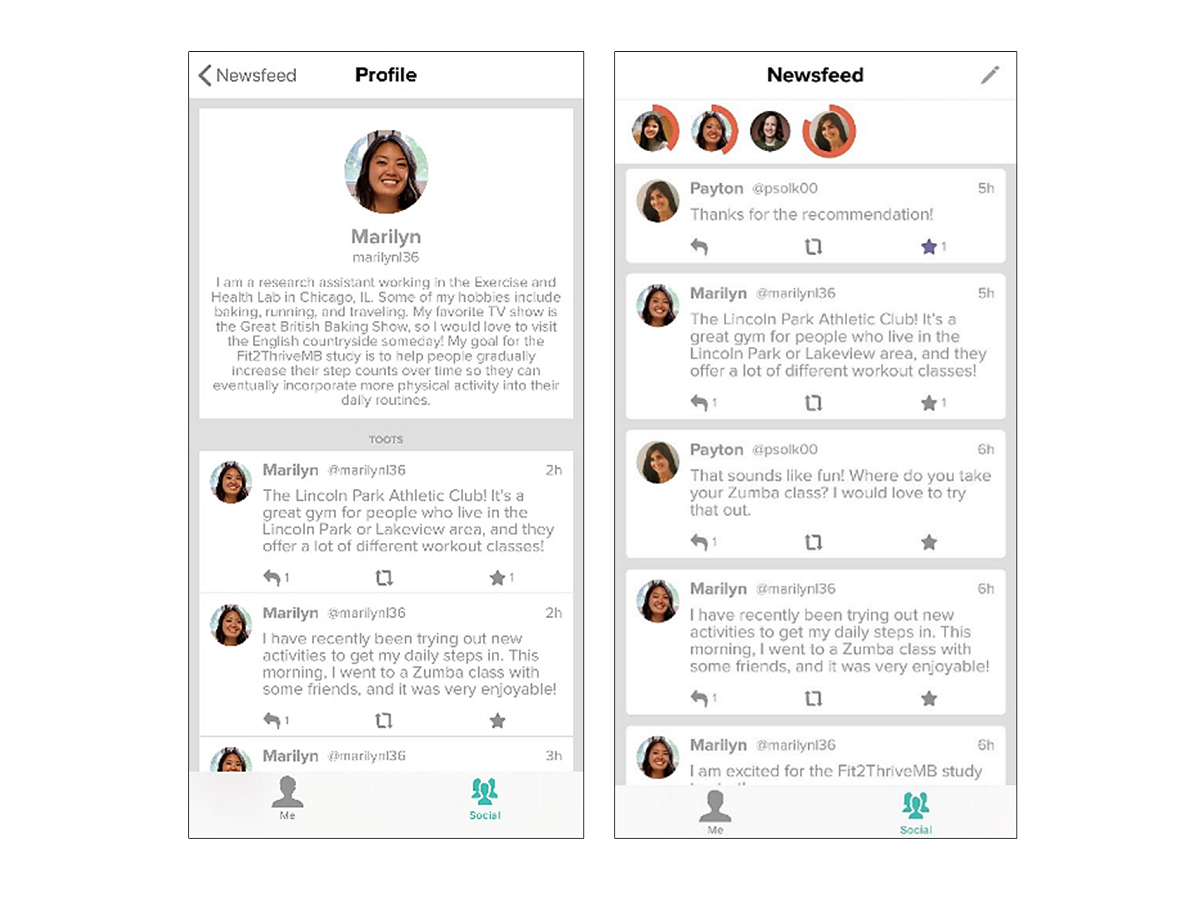
Fit2ThriveMB app social feed features.

This will consist of a message board where participants can communicate with 5 to 8 other participants in the intervention to provide encouragement and support. Participants will be randomized into groups using randomly permuted blocks. Each participant will be given their own social profile, with a short biography and optional picture. The social profile biographies include the following: hobbies, favorite physical activities, motivation to become more active, goals for participating in the Fit2ThriveMB program, and favorite quote or mantra. Participants will have the ability to write their own posts, comment on other people’s posts, and share photos and website links. Participants will also be able to view their group members’ relative progress toward their daily step goals, as a circle around each patient’s profile picture will fill in as they accumulate steps each day (Figure 5). Participants will receive notifications when someone posts in the social feed, when they have been tagged in a post, and when someone in their group has met their activity goal for the day. A study team member will act as a moderator and closely monitor and facilitate social feed to enhance use and prevent misuse. The study staff will also use social feed as a way to provide important updates about the Fit2ThriveMB app and Fitbit, postdiscussion questions, and provide links to cancer survivorship and PA educational resources (Textbox 2 for examples). During weeks 1-7 of the intervention, there will be 2 preplanned posts in the social feed. During weeks 8-12, the study staff will post 1 preplanned post a week in the social feed. Throughout the intervention, study staff will also post important updates regarding study information and logistics as needed, which may increase the total number of posts per week. The total time spent moderating and posting by the study staff will be approximately 30-40 minutes per week.

**Example Fit2ThriveMB social feed moderator posts.**

Physical activity discussion

- “What is your biggest barrier to becoming physically active? What are you trying to do to overcome this barrier? Reply to this post, and if you like another group member's response, ”favorite“ it by clicking on the star below!”
- “What are some of your favorite activities to get your daily steps in? Reply to this post and share with the group so everyone has some new ideas.”

Physical activity information and survivorship news and resources

- “If you’re trying to find new, different ways to get your steps in, click on the link below for some ideas as a jumping off point. Click on the ”star“ below to favorite this post if you are looking for new ideas or reply with some others that work for you! ”
- “If you are looking for events in the Chicago area, Susan G Komen has the following list of events for the 2020 year so far. ”Favorite“ this post if you are interested in attending one of these events or reply with other event ideas!”

Study information and logistics

- “Your week 3 assessment questionnaire was sent out today! Please complete the questionnaire as soon as possible.”
- “Activity monitors for your week 12 assessments will be mailed tomorrow. Keep an eye on your mail for the assessment packet. Keep up the great work in the homestretch!”

#### Coaching Calls

Participants will receive weekly coaching calls that will provide feedback on the previous week’s symptom burden and progress on PA goals, review personalized goals and strategies for increasing PA for the next week, inquire about any issues or injuries, and cover at least 1 SCT [22] behavior change strategy (Table 5 for topics covered). Participants will be instructed to call or email their coaches throughout the week if they encounter any issues. The coaching calls will be semistructured and recorded to ensure fidelity.

**Table 5:** Weekly coaching call topics for Fit2ThriveMB intervention group.

Week	Topic
1	Intro to coaching calls, getting started, and effective goal setting
2	Benefits of increasing physical activity and increasing steps
3	Benefits of reducing sitting or overcoming barriers to reducing sedentary behavior
4	Finding activities you enjoy
5	Self-monitoring
6	Managing symptoms
7	Overcoming barriers
8	Increasing self-efficacy
9	Realistic outcome expectations
10	Social support
11	Healthy rewards
12	Relapse prevention

### Technical Support

Participants will be provided with detailed instructions for setting up their Fitbit and Fit2ThriveMB app. Participants will be encouraged to contact study staff via phone, email, or the Fit2ThriveMB app social feed as soon as possible if any technical issues arise. All participants will be instructed to inform the study team immediately if they lose their Fitbit or if it malfunctions, and they will be sent a new device. They will also be asked about technical issues during their weekly coaching calls, which the study team will take note of, discuss with the technical support team, and follow up with the participant if needed.

### Privacy and Confidentiality

Participants will use a Quick Response code specific to them to log into the Fit2ThriveMB app. To protect their privacy, no personal information is stored or associated with their app account. All Fitbit and app data will be collected using the University server cluster, which has limited physical access, is firewalled, and regularly monitored for security issues. All phone encrypted data transmissions use a secure sockets layer protocol with a unique token for each participant. All data are backed up regularly, and any personal health information and Health Insurance Portability and Accountability Act data are stored on separate data clusters with unique keys and limited firewalled access. Participants will be informed of all potential privacy and confidentiality risks and practices in place to ensure protection in the informed consent form.

### Waitlist Attention Control: Healthy Lifestyle Arm

Participants assigned to this condition will be instructed to go about their usual activities. They will be provided with instructions for downloading and using the Cancer.Net app and encouraged to regularly use the Cancer.Net app features, including information about health and well-being, treatment guidelines specific to their cancer type, symptom tracking, and appointment tracking. Although the Cancer.Net app does provide content related to PA, this information is not prominent, and no information related to PA will be covered in this condition. To match Fit2ThriveMB contacts, participants will also receive weekly calls that will last approximately 10-15 minutes and cover health and well-being topics (Table 6) and be directed to content within the Cancer.Net app related to each topic. They will be provided with the Fitbit and Fit2ThriveMB app and all of its features, including the social feed, after completion of week 12 assessments and provided with a start date to begin receipt of the 12-week program. They will have the option to attend a brief program orientation with study staff and access to the Fit2ThriveMB app for the duration of the funding period (up to approximately 12 months).

**Table 6:** Weekly healthy lifestyle control group call topics.

Week	Topic
1	Mindfulness
2	Social support
3	Symptom tracking
4	Art therapy and doing and finding activities you enjoy
5	Sleep
6	Fatigue
7	Cognition
8	Hydration
9	Nutrition
10	Taste changes
11	Healthy grocery shopping
12	Stress management

### Adverse Event and Safety Monitoring

Safety will be measured based on the number and severity of adverse events reported. All participants will be instructed to report any adverse events to the study staff within 24 hours of the event. Participants will answer nonspontaneous adverse event questions at weeks 4, 8, and 12 to collect any information on any adverse events that may have occurred but were not reported to the study team.

### Data Analytic Plan

#### Power

Sample size calculations (n=50) were based on the successful completion rate defined as an attrition rate of ≤20% (ie, complete 12-week assessment) and adherence to daily goals and wearing Fitbit ≥70% (59/84) of study days. With half of the participants (n=25) assigned to each condition, there is >85% power to differentiate between a 60% (control) and 80% (Fit2ThriveMB) *successful completion* rate with a one-tailed, exact test for binomial proportion at a .05 significance level (SAS version 9.4).

#### Aim 1: Feasibility and Acceptability

Feasibility and acceptability will be analyzed using descriptive statistics (frequencies, means, and SDs). Feasibility will primarily be evaluated using the *successful completion rate* defined as ≥80% (40/50) of participants who enroll in the intervention will remain in the intervention and participants will meet their daily goal and wear Fitbit ≥70% (59/84) of study days. If a successful completion rate is achieved, Fit2ThriveMB will be deemed feasible. Additional measures of feasibility, fidelity, acceptability, and safety will also be summarized and considered with the *successful completion* rate before proceeding with a more definitive trial.

#### Aims 2 and 3: Examine Outcome Patterns for PA, Symptoms, Functional Performance, and QoL

We will employ an intent-to-treat approach by including all participants recruited in the study, regardless of compliance. Every effort will be made to collect all outcome measures, even if a participant does not engage in assigned treatments. Analysis of covariance will be used to compare study groups concerning mean changes in each outcome from baseline to 12 weeks with baseline adjustment. Data from these analyses will provide estimated means, SEs, and preliminary effect sizes for the Fit2ThriveMB intervention. These data will be used to identify a primary end point and further refine the Fit2ThriveMB intervention to be tested in a more definitive trial. We will also use regression models to explore the relationship between changes in each outcome and intervention adherence. This study is not designed to draw conclusions about Fit2ThriveMB efficacy without further study.

## Results

This study was funded in March 2019. Data collection began in February 2020 and ended in February 2021. A total of 49 women were randomized, and the results are expected to be published in the summer of 2021. Preliminary data indicate that the sample is 86% (n=42) White, 6% (n=3) African American, 6% (n=2) Asian, 4% (n=2) unknown (participants did not want to disclose their race), and 2% (n=1) other; 10% (n=5) of the sample identified as Hispanic or Latina.

## Discussion

### Principal Findings

Advances in the treatment of metastatic breast cancer have resulted in a growing population of women living with metastatic breast cancer [1]. However, the addition of years to life does not necessarily translate to quality years. Increasing PA is a potentially modifiable behavior that could attenuate the adverse physical and psychological health effects of living with metastatic breast cancer. Very few PA promotion interventions have been designed and tested in patients with metastatic breast cancer. In fact, most trials specifically exclude metastatic patients. Furthermore, many of the published trials on PA for patients with metastatic breast cancer require at least some on-site exercise sessions and have required significant adjustments to the planned exercise dose (ie, frequency and intensity) to accommodate the needs of metastatic patients. Thus, the feasibility and generalizability of these programs are limited.

### Limitations and Strengths

The Fit2ThriveMB study has several limitations. Our sample may not be representative of all patients with metastatic breast cancer because of the recruitment strategies employed and the requirements to use technology. Participants in this trial are likely to be more technologically savvy, motivated to change their PA behavior, and potentially healthier than the general patient population with metastatic breast cancer. Recruitment from a large academic medical center may also bias the sample in favor of White, more affluent, and highly educated participants. We actively recruited and enrolled diverse samples. However, diversity should be enhanced in the future, with larger studies by recruiting more broadly in community cancer centers, community organizations, and support groups. Another concern may be the potential for low intervention adherence. To prevent adherence problems, the significance of the project and participation expectations will be clearly stated during recruitment and orientation, which will be incentivized for the completion of assessments. Participants will be called weekly, reminders will be sent for all study-related milestones, and participants in the intervention group will be contacted if they do not wear the Fitbit for ≥7 days. In addition, we anticipate that the highly tailored nature of the intervention will significantly reduce attrition and increase adherence. We do not limit participation in this study based on the treatment status or characteristics that could impact activity levels. We anticipate that these variables will not differ between groups due to randomization, but we will conduct analyses to confirm this. If not, we will explore the potential impact of these factors on trial outcomes. Finally, we did not include a true no-contact control condition. Accumulated evidence indicates that remaining sedentary is unlikely to have any positive effect on our outcomes of interest in low-active patients with cancer. Our design is such that we will be able to compare the intervention condition with a control group that receives similar attention and an app to use but no PA promotion resources, which will allow us to delineate the effects of the intervention itself from the attention received from the study staff.

The Fit2ThriveMB trial was designed to determine the feasibility and acceptability of a 12-week technology-based PA promotion intervention in patients with metastatic breast cancer. We will also explore outcome patterns suggesting the efficacy of Fit2ThriveMB on PA, symptom burden, QoL, and functional performance. Despite the limitations noted above, the Fit2ThriveMB intervention has many potential advantages. First, this research will provide a better understanding of how to effectively promote PA in patients with metastatic breast cancer, an understudied population. Second, this is the first study to focus on increasing the cumulative daily PA of any intensity in patients with metastatic breast cancer. Previous studies have focused on high doses of more intense MVPA, which is not feasible for many patients with metastatic breast cancer. Light-intensity activity has been associated with health benefits, including the prevention of functional decline [10] and mortality [11] and improved QoL [11,12] and body composition [13]. This approach may have substantial health benefits for patients with metastatic breast cancer, facilitate gradual and safe PA adoption by enhancing mastery experiences, and be a more feasible behavioral target than high volumes of more strenuous activity [50,51]. Third, this is the first study to test a theory-based, technology-based PA promotion intervention in patients with metastatic breast cancer using the technology they already own. This approach allows for remote monitoring of patients and prevents many of the barriers associated with on-site programs (eg, travel, time constraints, or difficulties parking). Finally, this is the first study to adopt a highly tailored and individualized approach that takes into account the PA level and symptom burden in any patient population with cancer. This will likely increase adherence and uptake of the intervention by accounting for each survivor's capabilities.

### Conclusions

On the basis of the results of this study, we will determine the feasibility and acceptability of the Fit2ThriveMB intervention and examine outcome patterns that suggest efficacy. If the results indicate that Fit2ThriveMB is feasible and likely efficacious, the intervention will be refined to enhance usability and reflect changes in technology. We will then conduct a large-scale, fully powered trial of the entire Fit2ThriveMB intervention package on changes or maintenance of outcomes examined in this study and additional outcomes (eg, biomarker end points and fitness) in additional populations with advanced cancer. Ultimately, the proposed study will provide key evidence to support the feasibility, acceptability, and health benefits of increasing PA in patients with metastatic breast cancer using a scalable intervention strategy that could be easily integrated into care to improve health and disease outcomes.

## Abbreviations

mHealth: mobile health
MVPA: moderate-to-vigorous physical activity
PA: physical activity
QoL: quality of life
RCT: randomized controlled trial
SCT: social cognitive theory
SPPB: Short Physical Performance Battery
